# Ecological Assembly Processes Are Coordinated between Bacterial and Viral Communities in Fractured Shale Ecosystems

**DOI:** 10.1128/mSystems.00098-20

**Published:** 2020-03-17

**Authors:** R. E. Danczak, R. A. Daly, M. A. Borton, J. C. Stegen, S. Roux, K. C. Wrighton, M. J. Wilkins

**Affiliations:** a Earth and Biological Sciences Directorate, Pacific Northwest National Laboratory, Richland, Washington, USA; b Department of Soil and Crop Sciences, Colorado State University, Fort Collins, Colorado, USA; c Joint Genome Institute, Walnut Creek, California, USA; University of Tennessee at Knoxville

**Keywords:** hydraulic fracturing, microbial ecology, null modeling, shale, viral ecology

## Abstract

The ecological drivers that concurrently act upon both a virus and its host and that drive community assembly are poorly understood despite known interactions between viral populations and their microbial hosts. Hydraulically fractured shale environments provide access to a closed ecosystem in the deep subsurface where constrained microbial and viral community assembly processes can be examined. Here, we used metagenomic analyses of time-resolved-produced fluid samples from two wells in the Appalachian Basin to track viral and host dynamics and to investigate community assembly processes. Hypersaline conditions within these ecosystems should drive microbial community structure to a similar configuration through time in response to common osmotic stress. However, viral predation appears to counterbalance this potentially strong homogeneous selection and pushes the microbial community toward undominated assembly. In comparison, while the viral community was also influenced by substantial undominated processes, it assembled, in part, due to homogeneous selection. When the overall assembly processes acting upon both these communities were directly compared with each other, a significant relationship was revealed, suggesting an association between microbial and viral community development despite differing selective pressures. These results reveal a potentially important balance of ecological dynamics that must be in maintained within this deep subsurface ecosystem in order for the microbial community to persist over extended time periods. More broadly, this relationship begins to provide knowledge underlying metacommunity development across trophic levels.

**IMPORTANCE** Interactions between viral communities and their microbial hosts have been the subject of many recent studies in a wide range of ecosystems. The degree of coordination between ecological assembly processes influencing viral and microbial communities, however, has been explored to a much lesser degree. By using a combined null modeling approach, this study investigated the ecological assembly processes influencing both viral and microbial community structure within hydraulically fractured shale environments. Among other results, significant relationships between the structuring processes affecting both the viral and microbial community were observed, indicating that ecological assembly might be coordinated between these communities despite differing selective pressures. Within this deep subsurface ecosystem, these results reveal a potentially important balance of ecological dynamics that must be maintained to enable long-term microbial community persistence. More broadly, this relationship begins to provide insight into the development of communities across trophic levels.

## Abstract (precis)

Interactions between viral communities and their microbial hosts have been the subject of many recent studies in a wide range of ecosystems. The degree of coordination between ecological assembly processes influencing viral and microbial communities, however, has been explored to a much lesser degree. By using a combined null modeling approach, this study investigated the ecological assembly processes influencing both viral and microbial community structure within hydraulically fractured shale environments. Among other results, significant relationships between the structuring processes affecting both the viral and microbial community were observed, indicating that ecological assembly might be coordinated between these communities despite differing selective pressures. Within this deep subsurface ecosystem, these results reveal a potentially important balance of ecological dynamics that must be maintained to enable long-term microbial community persistence. More broadly, this relationship begins to provide insight into the development of communities across trophic levels.

## INTRODUCTION

The identification of ecological drivers that shape microbial communities is a challenge in many environments, where high microbial diversity, a lack of time-resolved samples, and variable geochemistry limit a full understanding of community assembly. Hydraulically fractured deep subsurface environments can act as model systems for investigating ecological principles governing assembly processes. The process of hydraulic fracturing involves high-pressure injection of water, chemical additives, and proppant (usually sand) into deep hydrocarbon-bearing shale formations to generate fracture networks and recover oil and gas resources. During this activity, diverse microbial communities of bacteria and archaea are inadvertently injected into the deep subsurface and exposed to high pressure and temperature and rapidly increasing salinity driven by the dissolution of shale-derived minerals (1). These consistent physicochemical pressures decrease microbial diversity by selecting for a subset of introduced halophilic microorganisms that are able to subsequently colonize this new fractured shale ecosystem and persist for extended periods of time (>350 days) (2–4). Microbial community dynamics are additionally influenced by the presence of abundant viral populations that drive both top-down control via predation of community members and bottom-up control on microbial activity through the release of cellular nutrients via cell lysis (3, 5). Importantly, the subsurface fracture network is essentially a closed system following the hydraulic fracturing process, allowing the study of time-resolved community dynamics in the absence of new microbial and viral inputs. Together, this information suggests that hydraulically fractured wells can serve as model systems to investigate constrained community assembly processes.

Microbial communities are shaped by different ecological assembly processes (Table 1) (6). First, two types of selection can lead to deterministic shifts within microbial communities (7). When some environmental pressure drives two communities to be significantly divergent, this observed difference is due to “variable selection.” Variable selection occurs when turnover between two communities is greater than expected by random chance and has been observed when communities experience varied geochemical conditions or organic matter composition (8, 9). In comparison, “homogeneous selection” occurs when some common stressor pushes two communities to be convergent (e.g., when turnover is lower than expected by random chance alone) (7). For example, microbial communities from a successional soil environment were driven to more similar configurations (10). Second, differences in organismal movement through space can significantly influence community structure. When organisms are capable of easily dispersing, “homogenizing dispersal” drives communities to be more similar than under “dispersal limited” scenarios, which are characterized by low organismal movement leading to communities which drift apart (7, 11). When comparing communities separated by time rather than space, “dispersal limitation” suggests that communities are changing due to drift rather than the inability to mix. Last, when no process dominates (e.g., there is moderate dispersal and weak selection), the turnover between communities is considered “undominated” (7, 11).

**TABLE 1:** Definition of terms used throughout the article

Term	Definition or explanation
βNTI	β-Nearest Taxon Index; a phylogenetic null model which can differentiate deterministic and stochastic processes.
RCBC	Raup-Crick (Bray-Curtis); a taxonomic null model which differentiates between different stochastic processes.
Variable selection	Occurs when selective pressures drive communities to divergent configurations (i.e., different geochemical conditions result in two communities being phylogenetically driven apart).
Homogenous selection	Occurs when selective pressures push communities toward a common composition (i.e., common osmotic stress drive two communities toward similar phylogenetic configurations).
Dispersal limitation	Indicates populations are unable to mix leading to development via ecological drift; stochastic process.
Homogenizing dispersal	Indicates two populations are capable of interactions, allowing members to freely exchange; stochastic process.
Undominated	Indicates that no single assembly process is capable of explaining variation (i.e., mixtures of processes occur).

Relative to the processes that drive microbial community assembly, the factors influencing viral community structure are poorly understood (12). The close association between viruses and their hosts could lead to viral community assembly, which mirrors those processes experienced by the hosts (12–14). For example, the viral community may assemble according to variable selection when environmental filtering drives highly dynamic host abundances. Conversely, if the microbial community is phylogenetically consistent through time, the viral community may be affected by “homogenous selection,” leading to convergent community composition. However, viruses are still separately subjected to environmental processes, such as grazing, salinity, UV exposure, or lifestyle (lytic versus lysogenic), which can complicate predictions (13–16). The interplay between these factors and viral community assembly is underexplored, partly due to the lack of universal marker genes within viruses which makes viral communities challenging to study (17). Using a combination of marker genes and reference-based metagenomic sequencing, one study used null models to attribute deterministic (“nonrandom”) assembly to a viral subcommunity from macaque feces but did not identify specific processes (18). Furthermore, the interplay between microbial and viral community assembly processes is completely unexplored, to our knowledge. By investigating the processes governing the assembly of paired microbial and viral communities and their coordination, we can better contextualize interactions across different trophic levels.

Hydraulically fractured shales provide a closed system to investigate potentially constrained ecological assembly processes affecting both microbial and viral communities. Within these shales, we expect that microbial communities would initially experience significant variable selection (e.g., will be driven to divergent configurations) due to the rapid environmental changes encountered upon introduction into the subsurface (e.g., a freshwater system transitioning to brine-level salinity). Once the community acclimates to shale conditions, however, we might expect that a common community configuration will be maintained due to strong homogeneous selection (barring external perturbations) because ill-adapted members would have been filtered out by high pressure, temperature, and salinity. Such pattern is observed in many fractured shale ecosystems where *Halanaerobium* sp. is consistently a dominant microbial community member in late-produced water samples (2, 3, 5). In addition to abiotic controls, Borton et al. (2018) demonstrated that numerous metabolic handoffs likely help maintain these microbial communities, which could further enhance homogenous selection due to increased ecological interconnections (8, 19, 20). Additional studies have revealed that viral predation could exert a significant top-down control on the persisting microbial communities that may also result in community composition being relatively consistent through time (1, 3, 5). Given these past observations and known environmental forcing driven by *in situ* physicochemical conditions (e.g., salinity), we expect the microbial community structure will be primarily shaped by homogeneous, deterministic processes after injection into the deep subsurface. In turn, we expect that viral communities will assemble according to similar processes encountered by the host microbial community due to their narrow host range (21, 22). Specifically, selection should first deterministically cause significant shifts in viral community composition of the input community (i.e., variable selection) and then constrain community composition to be consistent through time (i.e., homogeneous selection).

In this study, we applied the ecological null modeling tools β-nearest taxon index (βNTI) and Raup-Crick (Bray-Curtis) (RC_BC_), to investigate the ecological assembly processes affecting both microbial and viral populations in deep fractured shale ecosystems. We leveraged time-resolved metagenomic data sets across multiple shale wells and used ribosomal protein S3 (*rps3*) as a marker gene to infer microbial community assembly analyses. The recovery of viral sequences from the same data sets also allowed the same ecological null modeling tools to be applied to over 80% of the observed viral community, a proportion previously inaccessible to these methods through the use of viral marker genes. From these data, we show that the assembly processes that govern these two distinct communities are significantly related to each other, supporting their strong interdependence. These results further demonstrate the need to study assembly processes across multiple trophic levels simultaneously to fully understand factors driving spatiotemporal dynamics of any one trophic level.

## RESULTS AND DISCUSSION

### Viral predation counterbalances environmental filtering in governing microbial community assembly processes.

Samples collected from two hydraulically fractured wells in the Utica shale—termed Utica-2 (U-2) and Utica-3 (U-3)—are explored in Daly et al. in greater detail (5). Briefly, these wells exhibited geochemical characteristics similar to previously described fractured shales (3). Following hydraulic fracturing, produced waters from both wells became increasingly saline over time with U-2 fluids increasing from 1.3 g/liter to 172 g/liter total dissolved solids (TDSs) on day 302, and U-3 fluids increasing from 0.08 g/liter to 204 g/liter TDSs on day 159 (see Fig. S1 in the supplemental material). In order to measure the ecological assembly processes governing microbial community structure within these brine-like samples, two null modeling analyses, namely, β-nearest taxon index (βNTI) and Raup-Crick (Bray-Curtis) (RC_BC_) were performed. These approaches leverage randomized community structures to determine whether observed turnover between communities is higher or lower than would be expected by random chance. βNTI is a phylogenetic null model capable of distinguishing deterministic and stochastic assembly processes (23). When |βNTI| is greater than 2, deterministic processes have driven observed community differences. These deterministic processes can be further investigated. If βNTI is greater than 2, variable selection has driven the observed communities to be more dissimilar. Such environmental filtering has been observed when divergent geochemical conditions affected community composition (24). If βNTI is less than −2, homogenous selection pushed the observed communities toward a common configuration, as can be observed when river water exerts a potentially common set of stressors in some riverbeds (25, 26). When |βNTI| is less than 2, however, communities are as different as expected by random chance due to stochastic processes. In combination with the taxonomic null model RC_BC_, we can further distinguish these stochastic processes just as βNTI can distinguish deterministic processes (7, 23). If RC_BC_ is greater than 0.95, a decreased ability to mix has led to significant community drift leading a dispersal limitation signal. In contrast, when a system experiences significantly high exchange rates, a homogenizing dispersal signal (RC_BC_ of less than −0.95) can arise. Lastly, if |RC_BC_| is less than 0.95, no single assembly process was strong enough to exert control and thus an undominated signal would be observed (7). Given that RC_BC_ values are primarily useful when βNTI indicates stochastic processes are responsible for community differences, we only present RC_BC_ values when |βNTI| values are less than 2. By examining the dynamics of these two null models through space and time, we can investigate the ecological assembly processes affecting both the microbial and viral communities.

10.1128/mSystems.00098-20.1 FIG S1 Total dissolved solids (TDSs) within Utica-2 and Utica-3 through time. Download FIG S1, PDF file, 0.01 MB. Copyright © 2020 Danczak et al. 2020 Danczak et al. This content is distributed under the terms of the Creative Commons Attribution 4.0 International license.

**FIG 1:**
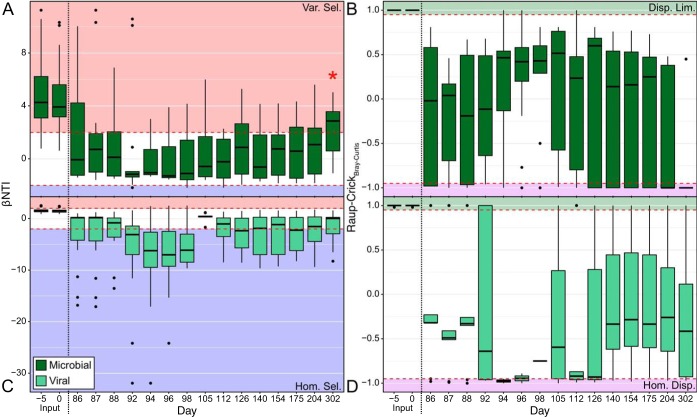
Ecological null modeling results for the Utica-2 samples through time; the −5 and 0 time points are samples collected from input fluids. (A) βNTI results for the microbial community; (B) RC_BC_ results for the microbial community; (C) βNTI results for the viral community; (D) RC_BC_ results for the viral community. Given that these are pairwise metrics, the boxplots represent values collected between the given time point and all others. In A and C, the red-shaded regions are those values interpreted as variable selection (βNTI of more than 2), the blue-shaded regions are those values interpreted as homogeneous selection (βNTI of less than −2), and the white regions indicate stochastic processes (|βNTI| of <2). In B and D, the green-shaded regions indicate dispersal limitation (RC_BC_ greater than 0.95), the purple represents homogeneous dispersal (RC_BC_ less than −0.95), and the white regions are undominated. Given RC_BC_ is only interpretable for stochastic βNTI results, only RC_BC_ values for insignificant βNTI comparisons are presented. The red asterisk/star indicates a discrepancy in interpretation between rarefied and nonrarefied data.

**FIG 2:**
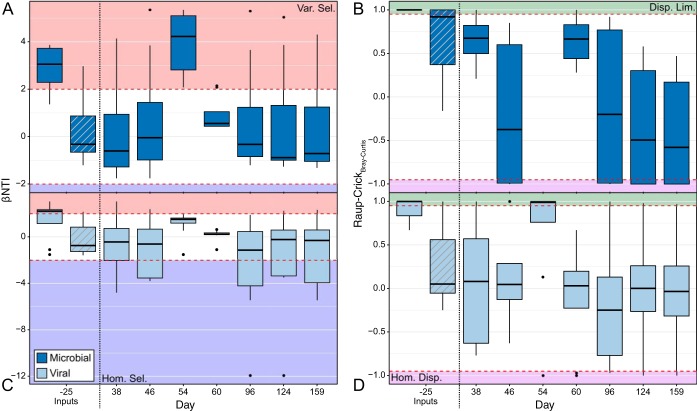
Ecological null modeling results for the Utica-3 samples through time; the −25 time point represents samples collected from the freshwater input fluid (solid) and the recycled input fluid (gray diagonal lines). (A) βNTI results for the microbial community; (B) RC_BC_ results for the microbial community; (C) βNTI results for the viral community; (D) RC_BC_ results for the viral community. Given that these are pairwise metrics, the boxplots represent values collected between the given time point and all others. In A and C, the red-shaded regions are those values interpreted as variable selection (βNTI greater than 2), the blue-shaded regions are those values interpreted as homogeneous selection (βNTI less than −2), and the white regions indicate stochastic processes (|βNTI| less than 2). In B and D, the green-shaded regions indicate dispersal limitation (RC_BC_ greater than 0.95), the purple represents homogeneous dispersal (RC_BC_ less than −0.95), and the white regions are undominated. Given RC_BC_ is only interpretable for stochastic βNTI results, only RC_BC_ values for insignificant βNTI comparisons are presented.

Input fluids for the hydraulic-fracturing process are frequently generated using local freshwater sources, with microbial communities distinct from those that subsequently colonize and persist in the deep shale ecosystem. Consistently, we observe that microbial communities in freshwater inputs were significantly different from those in produced fluids due to variable selection (βNTI of >2) (Fig. 1A and B, Fig. 2A and B). This variable selection was likely driven by the development of high-salinity conditions in the deep fractured shales caused by the dissolution of mineral phases. Recycled produced fluids (RTs) from an older hydraulically fractured well were used as one input source in well U-3 and differed from later time points, primarily through undominated processes. This indicates that upon injection, microbial RT communities from well U-3 were influenced by some combination of deterministic and stochastic processes whereby neither dominates. This result is likely due to the microbial community being preadapted for saline down-hole conditions in the recycled input water. By surviving in a similar habitat in a different shale well, the RT communities differ due to isolation but maintain similarities through common selective pressures, which, when combined, lead to an undominated signal.

As hypothesized above, intense environmental filtering should occur on produced fluid samples, due to the strong environmental pressures experienced within the fractured shale (i.e., salinity, temperature, and pressure). In contrast to our expectation that homogeneous selection would maintain a consistent community configuration through time, the majority of community comparisons in wells U-2 and U-3 were in the stochastic range (|βNTI| of <2) (Fig. 1A and 2A), with RC_BC_ results suggesting that undominated processes were the major driver of community assembly (Fig. 1B and 2B). An undominated signal typically consists of some mixture of weak assembly processes, such as weak selection combined with a moderate level of dispersal (7, 11). However, previously observed widespread viral predation within these fractured shale environments (3, 5) might be the primary driver for this undominated signal. This signal may arise from a “kill-the-winner” (KTW) scenario counter-acting otherwise strong homogeneous selection exerted by the hypersaline conditions (5, 12, 27). A KTW scenario could be influential via two potential mechanisms. First, KTW could result in variable selection by continuously driving microbial community turnover through the elimination of the most abundant community members (28). Under this mechanism, the lack of a dominant community assembly process might be the result of strong variable selection imposed by viral predation being canceled out by a strong homogeneous selective pressure imposed by high salinity. Alternatively, KTW could directly result in stochastic assembly processes in the absence of strong homogeneous selection (29). In this case, we expect that dominant organisms would still be deterministically targeted by predation, but the resulting niche space would be filled stochastically. This mixture of deterministic and stochastic processes is expected to lead to an undominated signal (7). Regardless of which of these KTW mechanisms is the most accurate, the absence of a strong signal for homogeneous selection, despite a strong homogenizing pressure (e.g., salinity), suggests that viral predation may run counter to potential environmental filtering in fractured shale ecosystems.

### Viral populations develop according to homogenous selection and undominated processes.

Given the dependence of viruses on their microbial hosts for reproduction, we hypothesize that viral communities would be influenced by similar assembly processes as those that affected the microbial community. In this case, variable selection will act upon input samples while undominated processes structure later time points. As with the microbial community, we analyzed the viral community by measuring both βNTI and RC_BC_. Contrary to our expectations, null modeling results indicated that viral communities in freshwater input fluids in both wells differed significantly from those in produced fluids due to a greater influence of dispersal limitation (Fig. 1C and D, Fig. 2C and D), while viral populations in the recycled produced fluids in well U-3 were different due to undominated forces (Fig. 2C and D). The comparative absence of variable selection indicates that changes in environmental conditions after injection did not influence viral communities as strongly as they influenced microbial communities. Instead, separation in time (and thus lack of physical mixing) combined with stochastic ecological drift in the community (leading to a signal of dispersal limitation) was the primary driver of initial community change.

Viral communities encountered assembly processes that did not strictly align with those experienced by the host microbial community. In the initial produced waters of U-2, viral communities appear to be controlled by undominated forces followed by a period of strong homogeneous selection (Fig. 1C and D) linked to the *Halanaerobium* sp.-dominated microbial community (see Fig. S2 in the supplemental material) (5). Although similar assembly patterns have been observed within a mammalian ecosystem (18), this is the first time that this methodology has been attempted on such a large (>80%) proportion of an observed viral community using viral sequences identified *de novo*. Moreover, this observation suggests that the relatedness index used to calculate βNTI actually contains evolutionarily relevant information because this homogeneity coincides with the dominance of *Halanaerobium* sp. (5). After this point, the viral community never reached a similarly homogenous state, instead being governed by either homogeneous selection or undominated processes (Fig. 1C and D). In contrast, well U-3 was primarily controlled by undominated processes throughout the sampling period, never reaching a comparably homogenous condition (Fig. 2C and D). While the inferred viral assembly processes deviate from our initial hypothesis, the variability in assembly processes acting upon viral and microbial communities could be driven by the low host diversity within these ecosystems. Low-diversity ecosystems could drive viral populations to be more closely related due to changes in microdiversity (i.e., strain-level fluctuations), rather than total diversity (i.e., along broad phylogenetic lineages) (12, 27, 30, 31).

10.1128/mSystems.00098-20.2 FIG S2 Stacked bar charts representing the taxonomic composition of microbial communities in Utica-2 and Utica-3 through time. Download FIG S2, PDF file, 0.01 MB. Copyright © 2020 Danczak et al. 2020 Danczak et al. This content is distributed under the terms of the Creative Commons Attribution 4.0 International license.

### The development of viral communities is intrinsically linked to the microbial community in fractured shale ecosystems.

**FIG 3:**
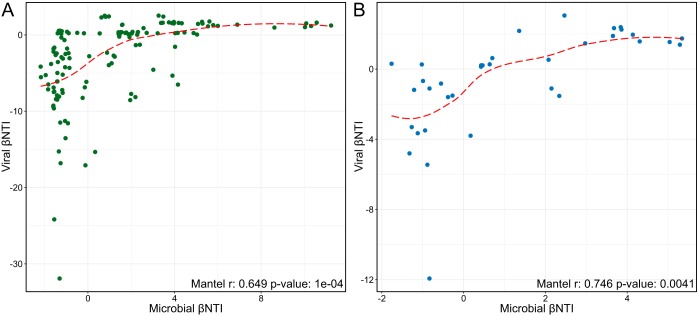
Correlations between microbial and viral βNTI values in wells U-2 (A) and U-3 (B). The red dashed line represents the Loess curve fit. In both panels, the Mantel correlation is presented in the bottom right.

While the specific community assembly processes experienced by the viral and microbial communities did not completely align, significant coordination appears to occur. To determine the relationship between the ecological processes acting upon viral and microbial communities, the null modeling results from both communities were correlated with each other using both a permuted Procrustes analyses and Spearman-based Mantel test (Fig. 3, see Table S2 in the supplemental material). Both the permuted Procrustes (U-2 m^2^ of 0.460, *P* = 0.0015; U-3 m^2^ of 0.479, *P*= 0.009) and Mantel tests (Fig. 3) revealed that viral and microbial βNTI results within each well were significantly correlated. This indicates that the deterministic processes shaping the phylogeny/relatedness of both communities are interlinked. Notably, the viral community appears to experience stronger homogenizing selection than the microbial community, as evidenced by low βNTI values (less than −30), likely related to a singular selective force primarily affecting viral structuring (i.e., host availability).

10.1128/mSystems.00098-20.4 TABLE S2 Table of Mantel-based statistics for comparisons between microbial and viral βNTI values. Download Table S2, XLSX file, 0.01 MB. Copyright © 2020 Danczak et al. 2020 Danczak et al. This content is distributed under the terms of the Creative Commons Attribution 4.0 International license.

While previous research has noted the connection between viral and microbial community dynamics (5, 13, 22, 32), this demonstrates that the ecological pressures structuring these communities are intrinsically linked; when one community experiences increased turnover due to shifts in selective pressures (indicated by higher βNTI), the other will likely experience it as well. These results potentially are the result of the dynamic feedback loop existing between the top-down and bottom-up effects a virus community exerts on the host microbial community, although an *in silico* analysis would be necessary for confirmation. The RC_BC_ Mantel tests provide further evidence of coordination in the assembly processes influencing the microbial and viral communities (U-2 r of 0.815, *P* = 0.0001; U-3 r of 0.745, *P* = 0.033) (Table S2). Collectively, our results indicate strong linkages in the ecological assembly processes affecting viral and microbial communities, although the underlying mechanisms leading to this coordination are not yet clear.

### Conclusions.

**FIG 4:**
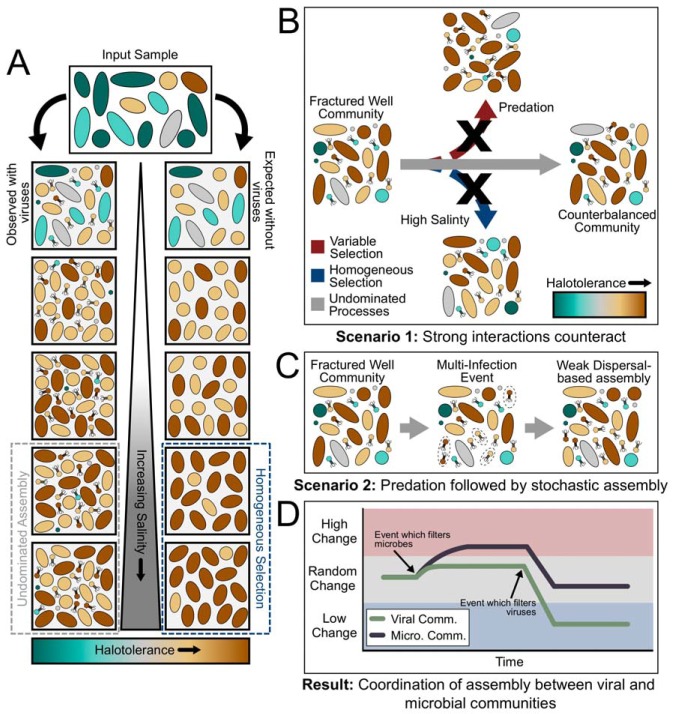
Conceptual model and potential explanations for observed microbial and viral community dynamics. (A) Contrast between expected and observed microbial dynamics. (B, C, and D) Detail the possible reasons for observed differences. (B) Within scenario 1, strong deterministic processes counterbalance, giving rise to undominated assembly for both the microbial and viral communities. (C) Within scenario 2, however, a multi-infection event leaves open niche space that is then occupied due to weak dispersal-based assembly processes, resulting in an undominated signal. (D) Regardless of the scenario, the result is the same, namely, coordination between the ecological assembly processes of microbial and viral communities. While the magnitudes might vary, these results suggest that one community will experience dynamics in response to the other.

While the interactions between viruses and their hosts in marine, soil, and subsurface environments have received increasing attention in recent years, no study to date has investigated the relationship between the ecological processes coordinating the assembly of each community. Here, we demonstrate that while each community is affected by different specific assembly processes at individual time points (Fig. 1 and 2), the overall assembly processes influencing these two communities are linked (Fig. 3). This supports past observations that across viral and host communities, fluctuations in one community lead to changes in the other (5, 13, 22). We suggest that widespread viral infection may act as a counterbalance to otherwise strong, homogenous environmental filtering, with the action of viral populations overriding strong deterministic factors from the abiotic environment (e.g., high salinity) that could otherwise lead to homogeneous selection (Fig. 4).

The interpretation of viral ecological modeling still poses a number of challenges. Viruses primarily have either a lysogenic or lytic lifestyle which could have ramifications for viral community development (12). Lysogenic viruses would likely experience different pressures than free lytic viruses given that they can integrate within host genomes until becoming virulent. For example, in a surface marine environment where high UV exposure may lead to free virus degradation, lysogenic phage could be protected and potentially more closely mirror microbial community dynamics (33). Once induced, however, lysogenic viruses might encounter lagged processes relative to their hosts, as observed within some marine environments (13). Alternatively, a community dominated by viruses with a lytic lifestyle might experience more divergent assembly than the microbial community (i.e., faster dispersal and inaccessibility to sufficient hosts). While the viral community within a fractured shale is a roughly equal mixture of these lifestyles (5), other ecosystems might have a different ratio of lysogenic and lytic viruses. Better quantification of viral lifestyles will help improve our understanding of the contributions that viruses make to ecological assembly processes. Another complication is that all of the viruses within this study were obtained via shotgun metagenomic sequencing and likely represent only a subset of the overall viral community (34). By collecting viromes, rather than metagenomes, a more complete viral community could be available, enabling us to better distinguish different viral groups (17, 34).

Beyond broader ecological considerations, these viral-host relationships have implications for the functioning of fractured shale ecosystems. Our results demonstrate that the two shale ecosystems examined here exhibit coordination between viral and microbial community assembly. Other studies have indicated that additional shale ecosystems develop along similar trajectories, although less information about viral community dynamics is available (2, 3, 35, 36). The apparent coordination and feedback between microbial and viral communities in the systems studied here suggest that such processes may be conserved across shale systems. If these feedback are disrupted, there may be impacts to the functioning of the shale ecosystem. For example, extensive viral predation could disrupt microbe-microbe interactions (e.g., between *Halanaerobium* sp. and *Methanohalophilus* sp.) and lead to changes in biogeochemical fluxes, such as the generation of new biogenic methane (19, 37). While hypothetical, these potential outcomes demonstrate the necessity of investigating how different assembly processes are partitioned within shale ecosystems. Furthermore, insights gained in this constrained shale ecosystem may be applicable to other environments that host more complex microbial and viral populations.

Overall, our results both confirm previous observations of the relationship between viral and microbial communities and demonstrate a strong association with respect to assembly processes, which has not been previously recognized. Not yet clear, however, are mechanisms that underlie coordination in assembly processes. To ultimately predict the effects of viral-host interplay across diverse ecosystems, such mechanisms need to be revealed through modeling efforts and lab-based experiments.

## MATERIALS AND METHODS

### Sample collection.

Hydraulic fracturing input fluids and shale-produced fluids were collected from wellheads and oil-gas-water separators in sterile bottles with no headspace (1 to 2 liters). When fluids were collected from the separator tanks, the separator tanks were flushed immediately before sample collection to minimize community changes due to incubation in the separator. Flow rates ranged from ∼400,000 liters per day at early time points to ∼170,000 liters per day at later time points, with a separator capacity of ∼5,500 liters. These fluids were collected from two wells in the Utica-Point Pleasant shale formation in Ohio (*n *= 2, Utica formation). Fluids were filtered on 0.22-μm pore size polyethersulfone filters (Millipore; Fisher Scientific). Produced fluids contained high concentrations of ferrous iron (100 to 180 mg liter^−1^). During the filtering process, the oxidizing iron resulted in a natural analog of the chemical flocculation protocol used to concentrate free viral particles (38). Thus, the viruses sampled in this study are probably a combination of free viral particles, integrated proviruses, and active, replicating viruses. Total nucleic acids were extracted from filters using lysis buffer I (39), purified with two phenol-chloroform and one chloroform-isoamyl alcohol extraction, and precipitated with NaCl and ice-cold ethanol. Nucleic acids were stored at –20°C until sequencing.

### Metagenomic sequencing and assembly.

Libraries were prepared using the Nextera XT library system according to manufacturer’s instructions. Sequencing adapters were ligated, and library fragments were amplified with 12 to 15 cycles of PCR before quantification with the KAPA Biosystems next-generation sequencing library kit. Following library preparation with a TruSeq paired-end cluster kit (v4), sequencing was performed on the Illumina HiSeq 2500 platform with HiSeq TruSeq sequencing by synthesis (SBS) sequencing kits (v4) following a 2 × 150-bp indexed run recipe. Fastq files were generated with CASSAVA 1.8.2. Fastq files were trimmed from the 5′ and 3′ ends with Sickle, and each sample was assembled individually with IDBA-UD using default parameters (5, 40, 41). Metagenome statistics, including the amount of sequencing are detailed in Table S1 in the supplemental material. Scaffolds of ≥5.0 kb were included in subsequent viral analyses.

10.1128/mSystems.00098-20.3 TABLE S1 Table containing the metagenome statistics. Download Table S1, XLSX file, 0.01 MB. Copyright © 2020 Danczak et al. 2020 Danczak et al. This content is distributed under the terms of the Creative Commons Attribution 4.0 International license.

### Microbial analysis.

An hidden Markov model (HMM) obtained from PFAM (PF00189) was utilized to find *rps3* sequences in each metagenomic assembly (42). Resulting sequences were clustered at 100% using the UCLUST option within USEARCH (43). Reads per kilobase million (RPKM) was calculated by mapping metagenomic reads to the clustered sequences using Bowtie2 and then normalizing read counts to sequencing depth (44). An alignment for these sequences was generated using MUSCLE and trimmed in Geneious to remove any position consisting of ≥95% gaps, after which a RAxML tree was generated using an evolutionary model approximated by PROTTEST (45–48) (see Text S1 in the supplemental material).

10.1128/mSystems.00098-20.5 TEXT S1 Maximum-likelihood *rps3* tree file. Download Text S1, TXT file, 0.03 MB. Copyright © 2020 Danczak et al. 2020 Danczak et al. This content is distributed under the terms of the Creative Commons Attribution 4.0 International license.

### Viral analysis.

Assemblies were mined for viruses using the methods outlined in Daly et al. (2019). Briefly, VirSorter was run using the “virome” database to identify viral sequences (49). Sequences resulting in category 1 or 2 status were retained and clustered into viral OTUs using the “ClusterGenomes” (v1.1.3) app in CyVerse, using 95% average nucleotide identity and 80% alignment fraction of the smallest contig (50). Length-normalized viral operational taxonomic unit (OTU) abundances were then determined by mapping reads to viral sequences using Bowtie2, dividing read counts by length, and adjusting individual abundances by summed values within a sample (5, 44). Due to the lack of marker genes across viruses, a protein clustering approach was utilized to generate a tree which approximates taxonomic relationships as follows. First, vConTACT was used to compare gene content across viral genomes/contigs and obtain all-versus-all pairwise similarity values (51). Viral sequences which shared no protein content were assigned a similarity score of 0 in order to allow their incorporation into the tree. These similarity values were then standardized between 0 and 1 in order to capture subtle differences which were masked by large scores. These similarity values were finally converted to dissimilarity values and used to generate a unweighted pair group method using average linkages (UPGMA) tree (*upgma* command, “phangorn” package v2.4.0) (see Text S2 in the supplemental material) (52).

10.1128/mSystems.00098-20.6 TEXT S2 UPGMA-clustering tree of vConTACT data (“viral tree”). Download Text S2, TXT file, 0.1 MB. Copyright © 2020 Danczak et al. 2020 Danczak et al. This content is distributed under the terms of the Creative Commons Attribution 4.0 International license.

### Ecological null modeling.

Ecological null modeling was performed in order to investigate the ecological drivers affecting both microbial and viral communities (7, 23, 53). Specifically, β-nearest taxon index (βNTI) and Raup-Crick (Bray-Curtis) (RC_BC_) were used to determine contributions from selective and dispersal-based processes, respectively. First, β-mean nearest taxon distance (βMNTD) was calculated for each possible pairwise comparison within either the microbial or viral communities (*comdistnt*, “picante” package v1.8) (54). By comparing these observed βMNTD values to those obtained from 999 community randomizations, βNTI was calculated according to Stegen et al. (7, 23). These βNTI results can then be used to investigate the phylogenetic turnover within both communities and to understand whether deterministic (i.e., selection) or stochastic (i.e., random) processes affected community composition. If a |βNTI| value exceeds 2, a deterministic process shaped the microbial or viral community; if a |βNTI| value is less than 2, a stochastic process affected the community. Deterministic processes can then be distinguished based upon the sign of the βNTI value. When βNTI is greater than 2, communities are significantly more different than would be explained by random chance due to variable selection. Such a process occurs when environmental conditions between the compared communities are different (e.g., differences in organic carbon quality), leading to variation in community composition (55). If βNTI is less than −2, communities are significantly more similar than would be expected by random chance due to homogeneous selection. This type of selection would occur when relatively spatiotemporally consistent environmental conditions drive communities toward a common configuration, such as in the case of microbial community succession in geochemically stable soil environments (10). Correlations between microbial and viral βNTI values were assessed using both a permuted Procrustes test (*protest* command, “vegan” package v2.5.4) and a Spearman-based Mantel test (*mantel* command, vegan package v2.5.4) (56).

In order to calculate βNTI, a phylogenetic tree is necessary. Given that metagenomic sequencing was primarily used to investigate the microbial communities, the *rps3* RAxML tree generated above was used to assess phylogenetic turnover. Rarefaction to the sample with the lowest sequencing depth was used to verify that results were not an artifact of sequencing depth, and any differences were noted. Viral null modeling was performed using the >80% of the viruses placed on the viral relationship tree following the methods detailed above. In some cases, different samples showed identical viral composition (i.e., the same viruses were present in some pairs of samples). While we would normally expect a homogeneous selection signal in comparisons between these communities, βMNTD calculations result in no nearest neighbor distance, as the nearest neighbor is identical in all cases. When calculating βNTI, this results in a divide-by-zero error, as the null models are also identical. In order to alleviate these problems, small amounts of nearest neighbor “noise” were added in the form of very small values (10^−5^) to the null βMNTD measurements to indicate that a null phylogenetic tree has slightly augmented distances.

In addition to βNTI calculations, RC_BC_ was used to further distinguish observed stochastic processes according to Stegen et al. (23). Using 9,999 iterations per pairwise comparison, null communities were probabilistically generated based upon observed microbial and viral abundances. From these communities, a null distribution of Bray-Curtis values was calculated and then compared to observed Bray-Curtis values. Resulting deviations of the observed values from the null expectation represent the RC_BC_ metric once normalized from +1 to −1. Significant RC_BC_ values (|RC_BC_| of >0.95) suggest that communities are governed by either dispersal limitation or homogenous dispersal. Dispersal limitation (RC_BC_ of >0.95) occurs when two communities are significantly different due to being physically unable to mix in either time or space, leading to significant ecological drift (random changes in organismal abundance). Homogenous dispersal (RC_BC_ of less than −0.95) happens when two communities are significantly more similar due to the ability to freely mix throughout a given environment. Lastly, if |RC_BC_| is less than 0.95, the differences between communities arose due to undominated processes where no single ecological pressure is able to exert a dominating effect (i.e., weak dispersal and weak selection) (7). Given that these results are only useful in distinguishing stochastic processes, RC_BC_ values are only presented for insignificant βNTI values (i.e., |βNTI| of <2).

### Plot generation and code availability.

The program R (v3.5.4) was used to perform statistical tests and plot generation (57). All data plots were generated using “ggplot2” (package v3.1.1) (58). R scripts used within this study are available at https://github.com/danczakre/ShaleViralEcology.

### Data availability.

The metagenomes used in this study are publicly available through JGI; accession numbers can be found in Table S1.

## References

[1] Borton MA, Hoyt DW, Roux S, Daly RA, Welch SA, Nicora CD, Purvine S, Eder EK, Hanson AJ, Sheets JM, Morgan DM, Wolfe RA, Sharma S, Carr TR, Cole DR, Mouser PJ, Lipton MS, Wilkins MJ, Wrighton KC 2018 Coupled laboratory and field investigations resolve microbial interactions that underpin persistence in hydraulically fractured shales. Proc Natl Acad Sci U S A 115:E6585–E6594. doi:10.1073/pnas.1800155115. 29941576 PMC6048472

[2] Davis JP, Struchtemeyer CG, Elshahed MS 2012 Bacterial communities associated with production facilities of two newly drilled thermogenic natural gas wells in the Barnett Shale (Texas, USA). Microb Ecol 64:942–954. doi:10.1007/s00248-012-0073-3. 22622766

[3] Daly RA, Borton MA, Wilkins MJ, Hoyt DW, Kountz DJ, Wolfe RA, Welch SA, Marcus DN, Trexler RV, MacRae JD, Krzycki JA, Cole DR, Mouser PJ, Wrighton KC 2016 Microbial metabolisms in a 2.5-km-deep ecosystem created by hydraulic fracturing in shales. Nat Microbiol 1:1–9. doi:10.1038/nmicrobiol.2016.146. 27595198

[4] Lipus D, Vikram A, Ross D, Bain D, Gulliver D, Hammack R, Bibby K 2017 Predominance and metabolic potential of Halanaerobium spp. in produced water from hydraulically fractured Marcellus Shale wells. Appl Environ Microbiol 83:e02659-16. doi:10.1128/AEM.02659-16. 28159795 PMC5377500

[5] Daly RA, Roux S, Borton MA, Morgan DM, Johnston MD, Booker AE, Hoyt DW, Meulia T, Wolfe RA, Hanson AJ, Mouser PJ, Moore JD, Wunch K, Sullivan MB, Wrighton KC, Wilkins MJ 2019 Viruses control dominant bacteria colonizing the terrestrial deep biosphere after hydraulic fracturing. Nat Microbiol 4:352–361. doi:10.1038/s41564-018-0312-6. 30510171

[6] Vellend M 2010 Conceptual synthesis in community ecology. Q Rev Biol 85:183–206. doi:10.1086/652373. 20565040

[7] Stegen JC, Lin X, Fredrickson J, Konopka AE 2015 Estimating and mapping ecological processes influencing microbial community assembly. Front Microbiol 6:370. doi:10.3389/fmicb.2015.00370. 25983725 PMC4416444

[8] Danczak RE, Johnston MD, Kenah C, Slattery M, Wilkins MJ 2018 Microbial community cohesion mediates community turnover in unperturbed aquifers. mSystems 3:e00066-18. doi:10.1128/mSystems.00066-18. 29984314 PMC6030547

[9] Stegen JC, Johnson T, Fredrickson JK, Wilkins MJ, Konopka AE, Nelson WC, Arntzen EV, Chrisler WB, Chu RK, Fansler SJ, Graham EB, Kennedy DW, Resch CT, Tfaily M, Zachara J 2018 Influences of organic carbon speciation on hyporheic corridor biogeochemistry and microbial ecology. Nat Commun 9:585. doi:10.1038/s41467-018-02922-9. 29422537 PMC5805721

[10] Dini-Andreote F, Stegen JC, van Elsas JD, Salles JF 2015 Disentangling mechanisms that mediate the balance between stochastic and deterministic processes in microbial succession. Proc Natl Acad Sci U S A 112:E1326–E1332. doi:10.1073/pnas.1414261112. 25733885 PMC4371938

[11] Zhou J, Ning D 2017 Stochastic community assembly: does it matter in microbial ecology? Microbiol Mol Biol Rev 81:e00002-17. doi:10.1128/MMBR.00002-17. 29021219 PMC5706748

[12] Breitbart M, Bonnain C, Malki K, Sawaya NA 2018 Phage puppet masters of the marine microbial realm. Nat Microbiol 3:754–766. doi:10.1038/s41564-018-0166-y. 29867096

[13] Brum JR, Hurwitz BL, Schofield O, Ducklow HW, Sullivan MB 2016 Seasonal time bombs: dominant temperate viruses affect Southern Ocean microbial dynamics. ISME J 10:437–449. doi:10.1038/ismej.2015.125. 26296067 PMC4737935

[14] Mojica KDA, Brussaard C 2014 Factors affecting virus dynamics and microbial host-virus interactions in marine environments. FEMS Microbiol Ecol 89:495–515. doi:10.1111/1574-6941.12343. 24754794

[15] Kukkaro P, Bamford DH 2009 Virus-host interactions in environments with a wide range of ionic strengths. Environ Microbiol Rep 1:71–77. doi:10.1111/j.1758-2229.2008.00007.x. 23765723

[16] Brum JR, Schenck RO, Sullivan MB 2013 Global morphological analysis of marine viruses shows minimal regional variation and dominance of non-tailed viruses. ISME J 7:1738–1751. doi:10.1038/ismej.2013.67. 23635867 PMC3749506

[17] Sullivan MB 2015 Viromes, not gene markers, for studying double-stranded DNA virus communities. J Virol 89:2459–2461. doi:10.1128/JVI.03289-14. 25540374 PMC4325738

[18] Anthony SJ, Islam A, Johnson C, Navarrete-Macias I, Liang E, Jain K, Hitchens PL, Che X, Soloyvov A, Hicks AL, Ojeda-Flores R, Zambrana-Torrelio C, Ulrich W, Rostal MK, Petrosov A, Garcia J, Haider N, Wolfe N, Goldstein T, Morse SS, Rahman M, Epstein JH, Mazet JK, Daszak P, Lipkin WI 2015 Non-random patterns in viral diversity. Nat Commun 6:8147. doi:10.1038/ncomms9147. 26391192 PMC4595600

[19] Borton MA, Daly RA, O'Banion B, Hoyt DW, Marcus DN, Welch S, Hastings SS, Meulia T, Wolfe RA, Booker AE, Sharma S, Cole DR, Wunch K, Moore JD, Darrah TH, Wilkins MJ, Wrighton KC 2018 Comparative genomics and physiology of the genus Methanohalophilus, a prevalent methanogen in hydraulically fractured shale. Environ Microbiol 20:4596–4611. doi:10.1111/1462-2920.14467. 30394652

[20] Herren CM, McMahon KD 2017 Cohesion: a method for quantifying the connectivity of microbial communities. ISME J 11:2426–2438. doi:10.1038/ismej.2017.91. 28731477 PMC5649174

[21] Paez-Espino D, Eloe-Fadrosh EA, Pavlopoulos GA, Thomas AD, Huntemann M, Mikhailova N, Rubin E, Ivanova NN, Kyrpides NC 2016 Uncovering Earth’s virome. Nature 536:425–430. doi:10.1038/nature19094. 27533034

[22] Weitz JS, Poisot T, Meyer JR, Flores CO, Valverde S, Sullivan MB, Hochberg ME 2013 Phage-bacteria infection networks. Trends Microbiol 21:82–91. doi:10.1016/j.tim.2012.11.003. 23245704

[23] Stegen JC, Lin X, Fredrickson JK, Chen X, Kennedy DW, Murray CJ, Rockhold ML, Konopka A 2013 Quantifying community assembly processes and identifying features that impose them. ISME J 7:2069–2079. doi:10.1038/ismej.2013.93. 23739053 PMC3806266

[24] Stegen JC, Konopka A, McKinley JP, Murray C, Lin X, Miller MD, Kennedy DW, Miller EA, Resch CT, Fredrickson JK 2016 Coupling among microbial communities, biogeochemistry, and mineralogy across biogeochemical facies. Sci Rep 6:30553. doi:10.1038/srep30553. 27469056 PMC4965824

[25] Saup CM, Bryant SR, Nelson AR, Harris KD, Sawyer AH, Christensen JN, Tfaily MM, Williams KH, Wilkins MJ 2019 Hyporheic zone microbiome assembly is linked to dynamic water mixing patterns in snowmelt-dominated headwater catchments. J Geophys Res Biogeosci 124:3269–3280. doi:10.1029/2019JG005189.

[26] Graham EB, Crump AR, Resch CT, Fansler S, Arntzen E, Kennedy DW, Fredrickson JK, Stegen JC 2017 Deterministic influences exceed dispersal effects on hydrologically-connected microbiomes. Environ Microbiol 19:1552–1567. doi:10.1111/1462-2920.13720. 28276134

[27] Thingstad TF, Vage S, Storesund JE, Sandaa R-A, Giske J 2014 A theoretical analysis of how strain-specific viruses can control microbial species diversity. Proc Natl Acad Sci U S A 111:7813–7818. doi:10.1073/pnas.1400909111. 24825894 PMC4040589

[28] Rodriguez-Brito B, Li LL, Wegley L, Furlan M, Angly F, Breitbart M, Buchanan J, Desnues C, Dinsdale E, Edwards R, Felts B, Haynes M, Liu H, Lipson D, Mahaffy J, Martin-Cuadrado AB, Mira A, Nulton J, Pašić L, Rayhawk S, Rodriguez-Mueller J, Rodriguez-Valera F, Salamon P, Srinagesh S, Thingstad TF, Tran T, Thurber RV, Willner D, Youle M, Rohwer F 2010 Viral and microbial community dynamics in four aquatic environments. ISME J 4:739–751. doi:10.1038/ismej.2010.1. 20147985

[29] Xue C, Goldenfeld N 2017 Coevolution maintains diversity in the stochastic “kill the winner” model. Phys Rev Lett 119:268101. doi:10.1103/PhysRevLett.119.268101. 29328693

[30] Thingstad TF, Pree B, Giske J, Våge S 2015 What difference does it make if viruses are strain-, rather than species-specific? Front Microbiol 6:320. doi:10.3389/fmicb.2015.00320. 25941522 PMC4403507

[31] Ignacio-Espinoza JC, Ahlgren NA, Fuhrman JA 2019 Long-term stability and Red Queen-like strain dynamics in marine viruses. Nat Microbiol 5:265–271. doi:10.1038/s41564-019-0628-x. 31819214

[32] Brum JR, Ignacio-Espinoza JC, Roux S, Doulcier G, Acinas SG, Alberti A, Chaffron S, Cruaud C, de Vargas C, Gasol JM, Gorsky G, Gregory AC, Guidi L, Hingamp P, Iudicone D, Not F, Ogata H, Pesant S, Poulos BT, Schwenck SM, Speich S, Dimier C, Kandels-Lewis S, Picheral M, Searson S, Tara Oceans Coordinators, Bork P, Bowler C, Sunagawa S, Wincker P, Karsenti E, Sullivan MB 2015 Patterns and ecological drivers of ocean viral communities. Science 348:1261498. doi:10.1126/science.1261498. 25999515

[33] Koch AL 2007 Evolution of temperate pathogens: the bacteriophage/bacteria paradigm. Virol J 4:121. doi:10.1186/1743-422X-4-121. 17996103 PMC2169209

[34] Roux S, Adriaenssens EM, Dutilh BE, Koonin EV, Kropinski AM, Krupovic M, Kuhn JH, Lavigne R, Brister JR, Varsani A, Amid C, Aziz RK, Bordenstein SR, Bork P, Breitbart M, Cochrane GR, Daly RA, Desnues C, Duhaime MB, Emerson JB, Enault F, Fuhrman JA, Hingamp P, Hugenholtz P, Hurwitz BL, Ivanova NN, Labonté JM, Lee K-B, Malmstrom RR, Martinez-Garcia M, Mizrachi IK, Ogata H, Páez-Espino D, Petit M-A, Putonti C, Rattei T, Reyes A, Rodriguez-Valera F, Rosario K, Schriml L, Schulz F, Steward GF, Sullivan MB, Sunagawa S, Suttle CA, Temperton B, Tringe SG, Thurber RV, Webster NS, Whiteson KL, Wilhelm SW, Wommack KE, Woyke T, Wrighton KC, Yilmaz P, Yoshida T, Young MJ, Yutin N, Allen LZ, Kyrpides NC, Eloe-Fadrosh EA 2019 Minimum Information about an Uncultivated Virus Genome (MIUViG). Nat Biotechnol 37:29–37. doi:10.1038/nbt.4306. 30556814 PMC6871006

[35] Cluff MA, Hartsock A, Macrae JD, Carter K, Mouser PJ 2014 Temporal changes in microbial ecology and geochemistry in produced water from hydraulically fractured marcellus shale gas wells. Environ Sci Technol 48:6508–6517. doi:10.1021/es501173p. 24803059

[36] Murali Mohan A, Hartsock A, Bibby KJ, Hammack RW, Vidic RD, Gregory KB 2013 Microbial community changes in hydraulic fracturing fluids and produced water from shale gas extraction. Environ Sci Technol 47:13141–13150. doi:10.1021/es402928b. 24088205

[37] Graham EB, Stegen JC 2017 Dispersal-based microbial community assembly decreases biogeochemical function. Processes 5:65. doi:10.3390/pr5040065.

[38] John SG, Mendez CB, Deng L, Poulos B, Kauffman AKM, Kern S, Brum J, Polz MF, Boyle EA, Sullivan MB 2011 A simple and efficient method for concentration of ocean viruses by chemical flocculation. Environ Microbiol Rep 3:195–202. doi:10.1111/j.1758-2229.2010.00208.x. 21572525 PMC3087117

[39] Lever MA, Torti A, Eickenbusch P, Michaud AB, Å Antl-Temkiv T, Jã¸Rgensen BB 2015 A modular method for the extraction of DNA and RNA, and the separation of DNA pools from diverse environmental sample types. Front Microbiol 6:476. doi:10.3389/fmicb.2015.00476. 26042110 PMC4436928

[40] Peng YY, Leung HCM, Yiu SM, Chin F 2012 IDBA-UD: a de novo assembler for single-cell and metagenomic sequencing data with highly uneven depth. Bioinformatics 28:1420–1428. doi:10.1093/bioinformatics/bts174. 22495754

[41] Joshi N, Fass J 2011 Sickle: a sliding-window, adaptive, quality-based trimming tool for FastQ files. 1.33. https://github.com/najoshi/sickle.

[42] Eddy S, Wheeler T 2013 HMMER 3.1.

[43] Edgar RC 2010 Search and clustering orders of magnitude faster than BLAST. Bioinformatics 26:2460–2461. doi:10.1093/bioinformatics/btq461. 20709691

[44] Langmead B, Salzberg SL 2012 Fast gapped-read alignment with Bowtie 2. Nat Methods 9:357–359. doi:10.1038/nmeth.1923. 22388286 PMC3322381

[45] Edgar RC 2004 MUSCLE: multiple sequence alignment with high accuracy and high throughput. Nucleic Acids Res 32:1792–1797. doi:10.1093/nar/gkh340. 15034147 PMC390337

[46] Darriba D, Taboada GL, Doallo R, Posada D 2011 ProtTest 3: fast selection of best-fit models of protein evolution. Bioinformatics 27:1164–1165. doi:10.1093/bioinformatics/btr088. 21335321 PMC5215816

[47] Stamatakis A 2014 RAxML version 8: a tool for phylogenetic analysis and post-analysis of large phylogenies. Bioinformatics 30:1312–1313. doi:10.1093/bioinformatics/btu033. 24451623 PMC3998144

[48] Kearse M, Moir R, Wilson A, Stones-Havas S, Cheung M, Sturrock S, Buxton S, Cooper A, Markowitz S, Duran C, Thierer T, Ashton B, Meintjes P, Drummond A 2012 Geneious Basic: an integrated and extendable desktop software platform for the organization and analysis of sequence data. Bioinformatics 28:1647–1649. doi:10.1093/bioinformatics/bts199. 22543367 PMC3371832

[49] Roux S, Enault F, Hurwitz BL, Sullivan MB 2015 VirSorter: mining viral signal from microbial genomic data. PeerJ 3:e985. doi:10.7717/peerj.985. 26038737 PMC4451026

[50] Roux S, Brum JR, Dutilh BE, Sunagawa S, Duhaime MB, Loy A, Poulos BT, Solonenko N, Lara E, Poulain J, Pesant S, Kandels-Lewis S, Dimier C, Picheral M, Searson S, Cruaud C, Alberti A, Duarte CM, Gasol JM, Vaqué D, Tara Oceans Coordinators, Bork P, Acinas SG, Wincker P, Sullivan MB 2016 Ecogenomics and potential biogeochemical impacts of globally abundant ocean viruses. Nature 537:689–693. doi:10.1038/nature19366. 27654921

[51] Bolduc B, Jang HB, Doulcier G, You Z-Q, Roux S, Sullivan MB 2017 vConTACT: an iVirus tool to classify double-stranded DNA viruses that infect Archaea and Bacteria. PeerJ 5:e3243. doi:10.7717/peerj.3243. 28480138 PMC5419219

[52] Schliep KP 2011 phangorn: phylogenetic analysis in R. Bioinformatics 27:592–593. doi:10.1093/bioinformatics/btq706. 21169378 PMC3035803

[53] Stegen JC, Lin X, Konopka AE, Fredrickson JK 2012 Stochastic and deterministic assembly processes in subsurface microbial communities. ISME J 6:1653–1664. doi:10.1038/ismej.2012.22. 22456445 PMC3498916

[54] Kembel SW, Cowan PD, Helmus MR, Cornwell WK, Morlon H, Ackerly DD, Blomberg SP, Webb CO 2010 Picante: R tools for integrating phylogenies and ecology. Bioinformatics 26:1463–1464. doi:10.1093/bioinformatics/btq166. 20395285

[55] Stegen JC, Fredrickson JK, Wilkins MJ, Konopka AE, Nelson WC, Arntzen EV, Chrisler WB, Chu RK, Danczak RE, Fansler SJ, Kennedy DW, Resch CT, Tfaily M 2016 Groundwater-surface water mixing shifts ecological assembly processes and stimulates organic carbon turnover. Nat Commun 7:11237. doi:10.1038/ncomms11237. 27052662 PMC4829693

[56] Oksanen J, Blanchet FG, Friendly M, Kindt R, Legendre P, McGlinn D, Minchin PR, O’Hara RB, Simpson GL, Solymos P, Stevens MHH, Szoecs E, Wagner H 2019 vegan: community ecology package.

[57] R Development Core Team. 2011 R: a language and environment for statistical computing. R Foundation for Statistical Computing, Vienna, Austria.

[58] Wickham H 2016 ggplot2: elegant graphics for data analysis. Springer-Verlag, New York, NY.

