# Supplementary figures and images for: Ecological Assembly Processes Are Coordinated between Bacterial and Viral Communities in Fractured Shale Ecosystems
Source: mSystems. 2020 Mar 17;5(2):e00098-20. doi: 10.1128/mSystems.00098-20 (PMC7380583; doi:10.1128/mSystems.00098-20)

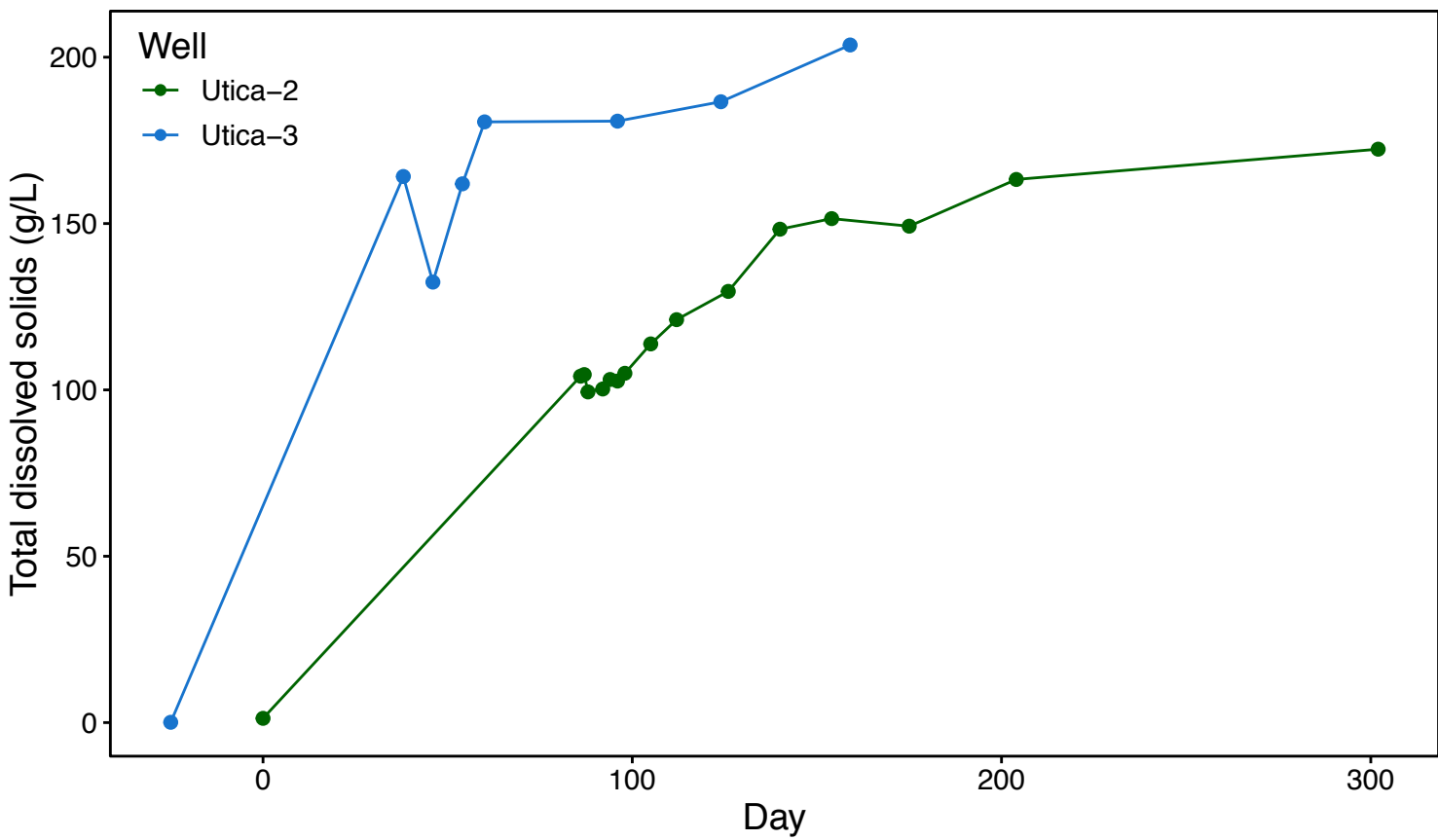

Supplement: FIG S1 [file mSystems.00098-20-sf001.pdf]

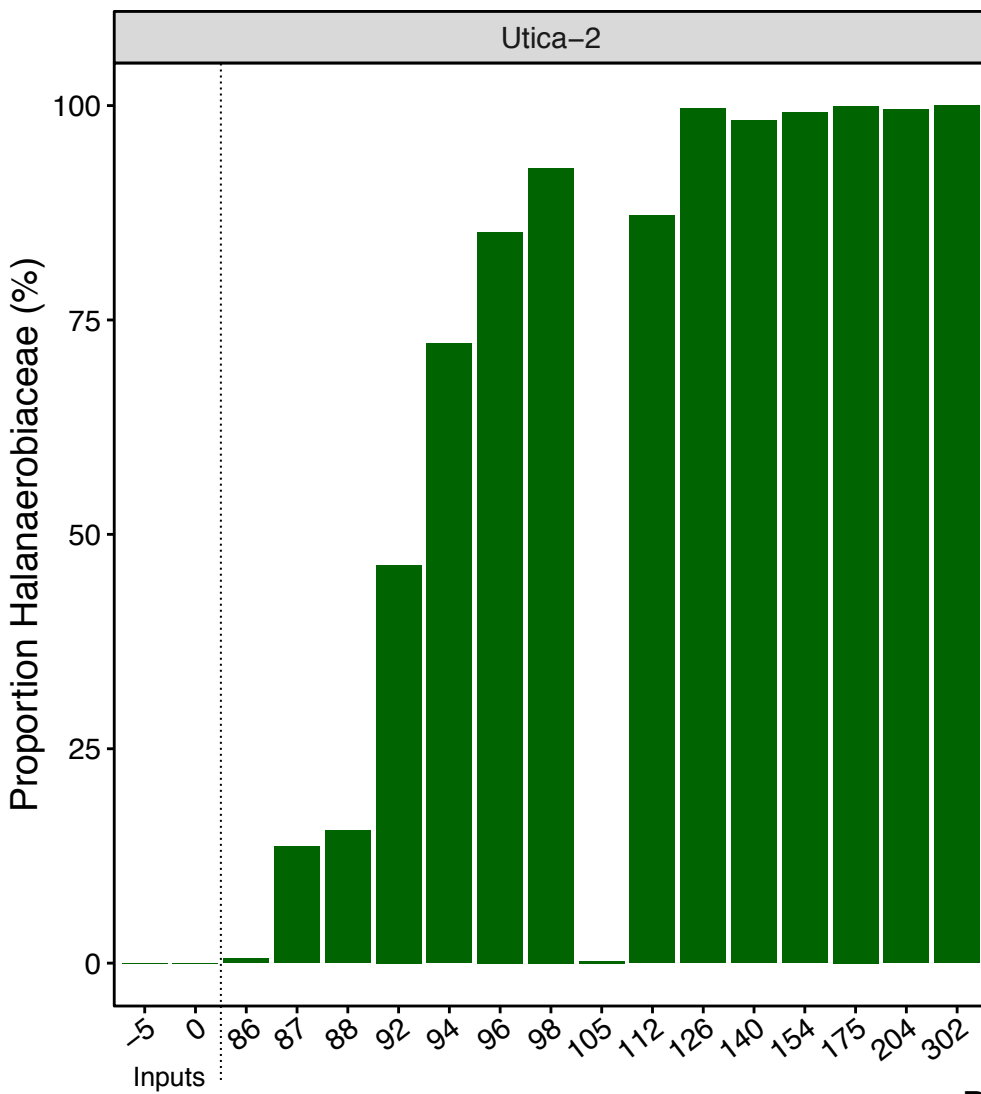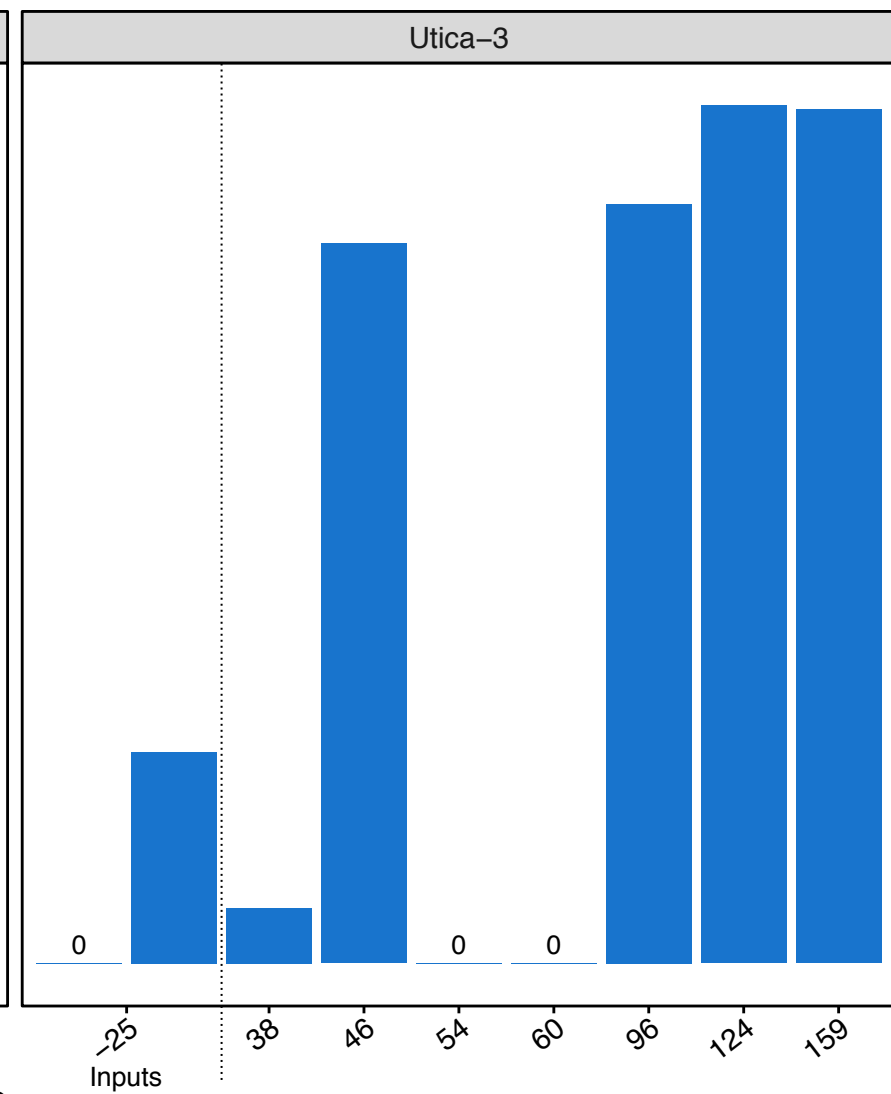

Day

Supplement: FIG S2 [file mSystems.00098-20-sf002.pdf]
